# Less biomass and intracellular glutamate in anodic biofilms lead to efficient electricity generation by microbial fuel cells

**DOI:** 10.1186/s13068-019-1414-y

**Published:** 2019-04-01

**Authors:** Daisuke Sasaki, Kengo Sasaki, Yota Tsuge, Akihiko Kondo

**Affiliations:** 1 Graduate School of Science, Technology and Innovation, Kobe University, 1-1 Rokkodaicho, Nada-ku, Kobe, Hyogo 657-8501 Japan; 2 Institute for Frontier Science Initiative, Kanazawa University, Kakuma-machi, Kanazawa, Ishikawa 920-1192 Japan; 3 RIKEN Center for Sustainable Resource Science, 1-7-22 Suehiro-cho, Tsurumi-ku, Yokohama, Kanagawa 230-0045 Japan

**Keywords:** Microbial fuel cell, Microbial community, Anodic biofilm, Biomass, *Geobacter*, Intracellular glutamate

## Abstract

**Background:**

Using a microbial fuel cell (MFC), we observed that a complex microbial community decomposed starch and transferred electrons to a graphite felt anode to generate current. In spite of the same reactor configuration, inoculum, substrate, temperature, and pH, MFCs produced different current and power density. To understand which factor(s) affected electricity generation, here, we analyzed a complex microbial community in an anodic biofilm and fermentation broth using Illumina MiSeq sequencing and metabolomics.

**Results:**

Microbial biomass on the anode was lower in MFCs generating more electricity (0.09–0.16 mg cm^−2^-anode) than in those generating less electricity (0.60–2.80 mg cm^−2^-anode), while being equal (3890–4196 mg L^−1^-broth) in the fermentation broth over the same operational period. Chemical oxygen demand removal and acetate concentration were also similar in fermentation broths. MFCs generating more electricity had relatively more exoelectrogenic bacteria, such as *Geobacter* sp., but fewer acetate-utilizing *Methanosarcina* sp. and/or *Lactococcus* sp. in anodic biofilms. Accordingly, anodic biofilms generating more electricity presented higher levels of most intracellular metabolites related to the tricarboxylic acid cycle and a higher intracellular ATP/ADP ratio, but a lower intracellular NADH/NAD^+^ ratio. Moreover, the level of intracellular glutamate, an essential metabolite for microbial anabolic reactions, correlated negatively with current density.

**Conclusion:**

Microbial growth on the anode and intracellular glutamate levels negatively affect electricity generation by MFCs. Reduced formation of anodic biofilm, in which intracellular glutamate concentration is 33.9 μmol g-cell^−1^ or less, favors the growth of acetate-utilizing *Geobacter* sp. on the anode and improves current generation.

**Electronic supplementary material:**

The online version of this article (10.1186/s13068-019-1414-y) contains supplementary material, which is available to authorized users.

## Background

Microbial fuel cells (MFCs) represent a promising technology for harvesting electrical energy from organic material in waste streams through the catalytic action of microorganisms [1–3]. Typically, the MFC contains wastewater as an environmentally friendly and complex substrate, while the microbial community of a mixed culture is inoculated into the anode chamber [4]. Anaerobic operation of the anode chamber results in the predominance of fermentative bacteria that convert complex substrates into short-chain fatty acids such as acetate, whereas electrogenic bacteria metabolize the fermentation products by electron transfer to the electrode [5, 6]. Electrogenic bacteria such as *Geobacter* sp. are capable of direct electron transfer to the anode without an external electron mediator and are found in contact with the anode in the biofilm [7]. Therefore, for a successful initiation of the MFC process, it is necessary to achieve efficient biofilm formation and maximize electron transfer on the anode [8]. Factors such as the inoculum, substrate, operational conditions (temperature, pH, and hydraulic-retention time), and reactor configuration (design, electrode materials, and cathode reaction) can affect the initiation process [9]. However, even when the above factors are the same, MFCs seem to generate different amounts of electricity. This observation suggests that one or more of these factors may be key to maximizing electricity generation.

The recent development of high-throughput sequencing of the 16S rRNA gene allows for high-resolution analysis of microbial community structure in MFCs [10]. For example, exoelectrogenic bacteria belonging to the genera *Geobacter* and *Desulfuromonas* were detected at a high frequency in acetate-fed biofilms on flame-oxidized stainless-steel anodes [11]. In addition, metabolomics can reveal the metabolic profile of a cell and infer putative microbial activities [12]. We previously used metabolomics to analyze electron transfer activity in *Geobacter sulfurreducens* at different poised potentials [13], as well as to study the microbial community on the surface of the MFC anode under different pH conditions [14]. These studies revealed that increased electricity generation correlated with higher intracellular flux through the tricarboxylic acid (TCA) cycle and ATP generation. Thus, approaches based on 16S rRNA gene sequencing and metabolomics could elucidate the structure and diversity of the complex microbial community in an MFC.

The aim of this study was to apply 16S rRNA gene sequencing and metabolomics to investigate the microbial community on the anodic electrode in air-cathode MFCs operated under the same conditions of inoculum, substrate, operational conditions, and reactor configuration, but generating different amounts of electricity. The ensuing results could help identify the factor(s) most likely to affect electricity generation in MFCs.

## Methods

### MFC configuration

The MFC reactor contained one cassette-electrode comprising an air–cathode, a separator, and an anode. Details of the reactor’s configuration have been described previously [14]. Carbon paper (TGP-H-120; MICLAB, Kanagawa, Japan) coated with 4-polytetrafluoroethylene layers and Pt-carbon (TEC10E70TPM; Tanaka Kikinzoku Kogyo, Tokyo, Japan) catalyst was used as the air–cathode. Graphite felt (F-203G; Sohgoh Carbon, Yokohama, Japan) and a glass filter (GF/A; GE Healthcare, Little Chalfont, UK) were utilized as the anode and separator, respectively. Four MFCs (MFC-1, MFC-2, MFC-1′, and MFC-2′) were operated, all with the same configuration.

### Operation of the MFCs

The composition of modified synthetic wastewater [15] was as follows: starch (1040 mg L^−1^), Bactopeptone (44.0 mg L^−1^), Bactoyeast extract (520 mg L^−1^), NH_2_Cl (116 mg L^−1^), KH_2_PO_4_ (28.6 mg L^−1^), CaCl_2_·2H_2_O (73.0 mg L^−1^), MgSO_4_·7H_2_O (1.40 mg L^−1^), KCl (70.0 mg L^−1^), NaHCO_3_ (29.0 mg L^−1^), and 3.30 mL L^−1^ of a trace-element solution (Deutsche Sammlung von Mikroorganismen und Zellkulturen medium 318; DSMZ, Braunschweig, Germany). MFCs were filled with the modified synthetic wastewater (pH 6.0). MFC operation was initiated by inoculating 20 mL activated sludge obtained from a sewage treatment plant operated by the Tokyo Metropolitan Government. Two MFCs, one with relatively high (MFC-1) and the other with relatively low (MFC-2) electricity generation, were operated. Then, the electrodes and inocula were changed, and the other two MFCs were operated, again generating more (MFC-1′) or less (MFC-2′) electricity. The modified synthetic wastewater was provided at a flow rate of 300 mL day^−1^ (hydraulic-retention time of 24 h) at an initial external resistance (*R*_ext_) of 10,000 Ω.

Voltage (*V*) produced by the MFC was monitored and recorded using a data logger (NR-1000; Keyence, Osaka, Japan), whereas current (*I*) and power density [*P* (mW m^−2^)] were calculated from the voltage at the set *R*_ext_ using the equations I=* V/R*_ext_ and *P *=* IV*, respectively. *R*_ext_ was dropped stepwise from 10,000 to 1000 Ω, 510 Ω, 200 Ω, 100 Ω, and 51 Ω, as the voltage increased in the MFCs.

### Analysis and calculation of MFC performances

Chemical oxygen demand (COD) was measured with a DRB200 system (Hach, Loveland, CO, USA) and COD removal efficiency (%) was calculated using the following equation:

1 $${\text{COD removal efficiency}} = \frac{{{\text{COD}}_{\text{in}} - {\text{COD}}_{\text{out}} }}{{{\text{COD}}_{\text{in}} }} \times 100\% ,$$

where COD_out_: COD in fermentation broth; COD_in_: COD in modified synthetic wastewater.

The average value of COD_in_ was 3139 ± 502 mg-COD L^−1^ (mean ± standard deviation). COD removal efficiency was measured several times during the final 10 days of operation in each MFC. To determine the suspended solids (SS), 1.0–5.0 mL of the suspended fraction was passed through a glass fiber membrane of 0.45 μm × 47 mm in diameter (Toyo Roshi Kaisha, Ltd., Tokyo, Japan), after which the membrane was dried at 105 °C for 120 min and then weighted.

The concentration of organic acids, such as acetate, propionate, butyrate, and lactate, was determined using a high-performance liquid chromatography system (HPLC; Shimadzu, Kyoto, Japan) equipped with a refractive index detector (RID-10A; Shimadzu) and an organic acid analysis column (Aminex HPX-87H; Bio-Rad Laboratories, Inc., Hercules, CA, USA). The HPLC was operated at 65 °C using 5 mM H_2_SO_4_ as the mobile phase with a flow rate of 0.6 mL min^−1^. Coulombic efficiency (C.E.) was calculated based on COD removal and the measured current, and assuming that 1 g of COD = 0.125 mol of electrons, 1 A = 5.39 × 10^23^ electrons per day, *F* = 96,485 C mol^−1^, and one electron = 1.60 × 10^−19^ C [16].

### Extraction of microbial genomic DNA

Microbial genomic DNA was extracted after MFC operation from the surface of the anodic electrode and fermentation broth as reported previously [17]. Purified DNA was eluted into TE buffer (10 mM TrisHCl, 1.0 mM EDTA) and stored at − 20 °C until use.

### Illumina library generation

Archaeal and bacterial 16S rRNA genes (V3–V4 region) were amplified using genomic DNA as template and primers Pro341F (5′-CCTACGGGNBGCWSCAG-3′) [18, 19] and Pro805R (5′-GACTACNVGGGTATCTAATCC-3′) [19]. Index primers (Nextera XT Index Kit; Illumina Inc., San Diego, CA, USA) overhanging the amplified sequences were added to the gene-specific sequences. PCR reactions and purification were performed according to the manufacturer’s instructions, and the purified amplicons were quantified as described previously [20]. The 16S rRNA genes along with an internal control (PhiX control v3; Illumina) were subjected to paired-end sequencing using the MiSeq next-generation sequencer, with a MiSeq reagent kit v3 (600 cycles; Illumina). Automated CASAVA 1.8 paired-end demultiplexed fastq were performed according to FASTQ Generation on the Illumina Basespace Sequence Hub (https://basespace.illumina.com/). Sequence quality control and feature table construction of the sequence data were performed and corrected by QIIME 2 version 2018.2 (https://qiime2.org) using the DADA2 pipeline [21]. The taxonomic composition of operational taxonomic units (OTUs) was classified via the Naive Bayes classifier. This classifier was trained on the Greengenes 13_8 99% OTUs full-length sequence database (https://data.qiime2.org/2018.2/common/gg-13-8-99-nb-classifier.qza). The OTU data were used for α-diversity estimation of the Faith’s Phylogenetic Diversity [22] and Shannon’s [23, 24] indices.

### Intracellular metabolite extraction and quenching

Cells were collected from the surface of the anode graphite felt after 30 days (MFC-1), 90 days (MFC-2), and 52 days (MFC-1′ and MFC-2′) of operation. Cell weight in each sample was adjusted to the same level based on optical density at 600 nm (OD_600_) before filtering each sample through a 4-polytetrafluoroethylene membrane filter (Omnipore, 0.45 µM, 47-mm diameter; Millipore, Billerica, MA, USA). The dry cell weight of each sample was estimated by multiplying the determined cell weight of *Escherichia coli* by OD_600_ using the following equation:

2 $${\text{Dry cell weight }}\left( {\text{mg}} \right) = 0.0582 \times {\text{OD}}_{600} \times {\text{cell suspension }}\left( {\upmu{\text{L}}} \right).$$

Immediately after filtration, the cells were washed with cold phosphate-buffered saline (PBS: 137 mM NaCl, 8.10 mM Na_2_HPO_4_, 2.68 mM KCl, 1.47 mM KH_2_PO_4_). Membrane filters with the washed cells were transferred to 50-mL centrifuge tubes and then frozen in liquid nitrogen. Metabolites were extracted from the cells using a modified cold chloroform-methanol method [25]. Finally, the water phase of the extract (700 µL) was dried under vacuum and stored at − 80 °C until further analysis [26].

### Mass spectrometry

The dried extract samples were thawed on ice, derivatized at 30 °C for 90 min with 100 µL of 20 mg mL^−1^ methoxyamine hydrochloride in pyridine, after which 50 µL *N*-methyl-*N*-(trimethylsilyl) trifluoroacetamide (GL Sciences, Tokyo, Japan) [27] was added followed by incubation at 37 °C for 30 min. Derivatized samples (1 µL) were subjected to gas chromatography-quadrupole-mass spectrometry (GC-Q-MS) using a GCMSQP-2010 system (Shimadzu) to detect metabolites from the TCA cycle, glutamate, and glucose.

Aliquots of the dried extract samples were also dissolved in 50 µL Milli-Q water and prepared for analysis by liquid chromatography-triple-stage quadrupole-mass spectrometry (LC-QqQ-MS) using an HPLC Agilent 1200 series for LC and Agilent 6460 with Jet Stream Technology for MS (Agilent Technologies, Waldbronn, Germany) controlled by MassHunter Workstation Data Acquisition software (v. B. 04.01; Agilent Technologies). The following compounds were detected: metabolites from the Embden-Meyerhof and pentose phosphate pathways, acetyl-CoA, ATP, ADP, nicotinamide adenine dinucleotide (NADH, NAD^+^), and nicotinamide adenine dinucleotide phosphate (NADPH, NADP^+^) [28]. Details of the GC-Q-MS and LC-QqQ-MS operating conditions and procedures have been described previously [29, 30]. Metabolite concentration was measured by triplicate sample injections.

### Bioinformatics and statistical analyses

The α-diversity indices (Faith’s Phylogenetic Diversity and Shannon’s index) were calculated using the QIIME 2 platform, because they best fit the data distribution. Faith’s Phylogenetic Diversity is the phylogenetic analogue of taxon richness [22].

Shannon’s index is commonly used to assess species diversity in a microbial community [23, 24]. The Kruskal-Wallis test was used to analyze COD removal efficiency and Student’s *t* test was applied to analyze C.E. and triplicate measurements of intracellular metabolites. Tests were performed using the JMP 13 software (SAS Institute Inc., Cary, NC, USA). *P *< 0.05 was considered statistically significant.

## Results

### MFCs exhibit high or low current generation

**Fig. 1:**
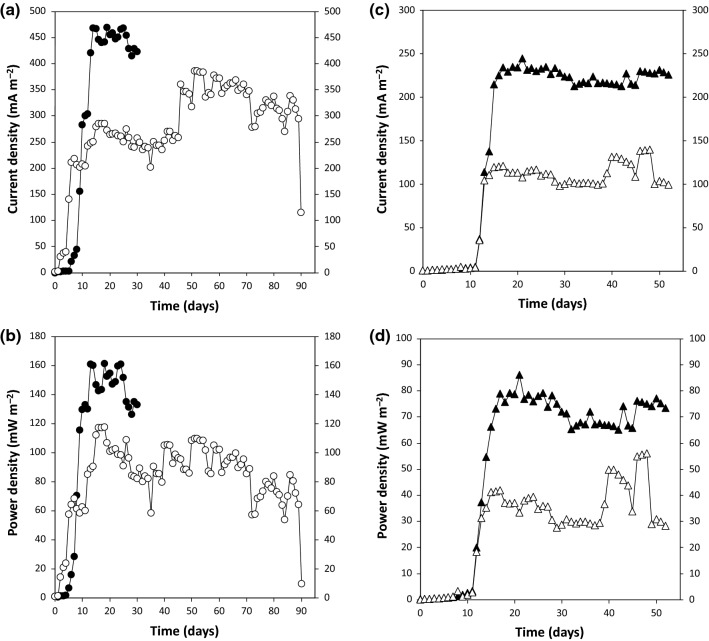
Average values of **a** current density (mA m^−2^) and **b** power density (mW m^−2^) in MFC-1 (closed circles) and MFC-2 (open circles), as well as **c** current density (mA m^−2^) and **d** power density (mW m^−2^) in MFC-1′ (closed triangles) and MFC-2′ (open triangles) during the operational period

Four MFCs with the same reactor configuration were loaded with modified synthetic wastewater at the same dilution rate. The inoculum was the same between MFC-1 and MFC-2, and between MFC-1′ and MFC-2′. The pH in the fermentation broth of MFCs was maintained at approximately pH 6.5 throughout the operational periods. Current density and power density in all MFCs increased until day 15 (Fig. 1).

Biochemical and electrochemical performances of MFC-1, MFC-2, MFC-1′, and MFC-2′ were compared (Table 1). COD removal efficiencies were similar between MFC-1 and MFC-2 (Kruskal-Wallis test, *P *= 0.083), and between MFC-1′ and MFC-2′ (*P* = 0.691). In contrast, *C.E.* values were higher in MFC-1 (or MFC-1′), compared to MFC-2 (or MFC-2′) (Student’s *t* test, *P *= 0.013 or 0.001), corresponding to the results of current generation (Fig. 1).

**Table 1:** Biochemical and electrochemical performance during stable operation of microbial fuel cells (MFCs)

MFC	COD removal efficiency (%)	SS anode (mg cm^−2^-anode)	SS broth (mg L^−1^-broth)	C.E. (%)
MFC-1	80.7 ± 8.30	0.09	1733	6.09 ± 0.55
MFC-2	87.1 ± 8.90	2.80	4491	3.70 ± 0.14
MFC-1′	78.2 ± 10.8	0.16	3890	3.31 ± 0.28
MFC-2′	80.6 ± 9.56	0.60	4196	1.57 ± 0.21

COD, chemical oxygen demand; SS, suspended solids; C.E., Coulombic efficiency

**Fig. 2:**
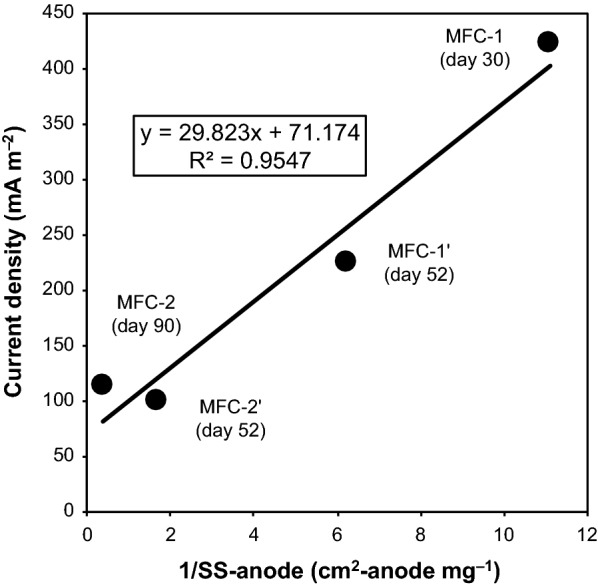
Correlation between current density and the inverse amount of suspended solids (SS) on the anode in four MFCs. A solid line and its equation indicate the best-fit linear relationship

SS were chosen as a rough estimate of the amount of microbial cells. At the end of the operation, the fermentation broth in MFC-2 contained more SS compared to that in MFC-1, probably due to a longer operational period (Table 1). By aligning the operational period, the fermentation broths of MFC-1′ and MFC-2′ yielded the same SS. Interestingly, SS on the surface of the anode correlated negatively with current generation (Fig. 2). In contrast, concentrations of organic acids and fermentation products were similar between different MFCs, particularly between MFC-1′ and MFC-2′, which shared the same operational period (Table 2). These results suggest that large biofilm formations on the anode negatively affect current generation, in spite of similar microbial growth and degradation activity between different MFCs.

**Table 2:** Concentrations of organic acids in the fermentation broths collected after 30 days and 90 days of operation of MFC-1 and MFC-2, respectively, and after 52 days of operation of MFC-1′ and MFC-2′

MFC	Organic acid (mM)			
	Acetate	Propionate	Butyrate	Lactate
MFC-1	1.97	0.61	1.47	ND
MFC-2	1.46	1.30	0.41	ND
MFC-1′	1.95	0.92	1.22	0.07
MFC-2′	1.99	0.95	1.52	0.06

ND, not detected

### Relative abundance of *Geobacter* sp. in anodic biofilms increases in MFCs with high current generation

The composition of the microbial community in the anodic biofilm and fermentation broth was analyzed by next-generation sequencing of prokaryotic 16S rRNA genes at the end of MFC operation. The number of observed OTUs and microbial diversity indices were higher in the anodic biofilm than in the fermentation broth for all four MFCs (Table 3 and Additional file 1).

**Table 3:** Summary of 16S rRNA gene sequencing data and α-diversity values (Faith’s Phylogenetic Diversity and Shannon’s index) in MFC-1 and MFC-2 after 30 and 90 days of operation, respectively

	Read counts	Observed OTUs	Faith’s Phylogenetic Diversity	Shannon’s index
MFC-1-anode	803,520	993	68.7	6.67
MFC-2-anode	880,770	947	69.2	7.24
MFC-1-broth	423,345	647	37.8	5.62
MFC-2-broth	458,184	520	27.2	5.40

OTUs, operational taxonomic units

**Fig. 3:**
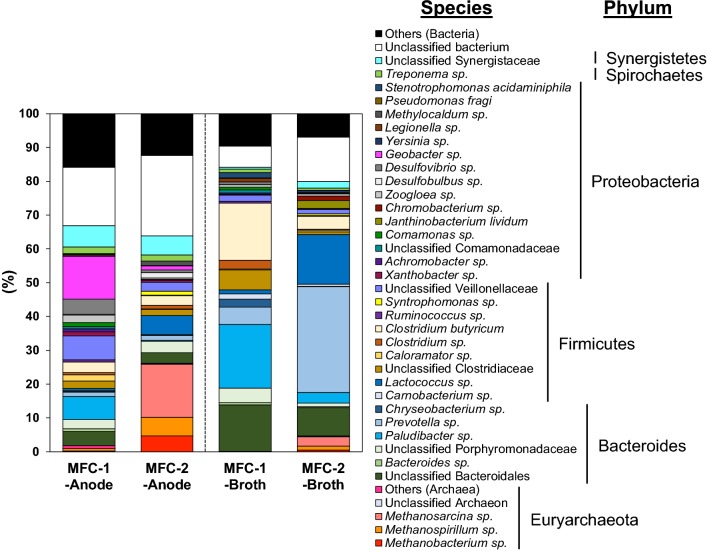
Species-level composition of archaea and bacteria in the anodic biofilm (Anode) and fermentation broth (Broth) of MFC-1 and MFC-2. Samples were obtained from MFC-1 and MFC-2 anodic biofilms and fermentation broths after 30 days and 90 days of operation, respectively. Not clustered (< 99% similarity) and low-abundance (< 1.0%) sequences were included in Unclassified and Others, respectively

Four major phyla, Euryarchaeota, Firmicutes, Bacteroidetes, and Proteobacteria were found in the anodic biofilm and fermentation broth of the four MFCs (Fig. 3 and Additional file 2). However, Euryarchaeota predominated in the anodic biofilm of MFC-2, which generated less electricity; whereas *Geobacter* sp. was predominant in the anodic biofilm of MFC-1, which generated relatively more electricity. Moreover, *Geobacter* sp. was more numerous in the MFC-1 anode than in the fermentation broths of both MFC-1 and MFC-2 (Fig. 3). Similarly, *Geobacter* sp. was more abundant in the anodic biofilm of MFC-1′, which generated more electricity, than in that of MFC-2′, which generated less electricity, and in the fermentation broths of both MFC-1′ and MFC-2′ (Additional file 2). Other dominant species in the anodic biofilm of MFC-1 included *Desulfovibrio*, *Clostridium*, *Lactococcus*, *Paludibacter*, and *Bacteroides*. However, a clear correlation between relative abundances of these bacterial species and electricity generation was not observed. On the other hand, the anodic biofilm and fermentation broth of MFC-2 exhibited higher levels of the methanogenic archaea *Methanosarcina*, *Methanospirillum*, and *Methanobacterium* spp. compared to all other MFCs.

### Intracellular glutamate is lower in the anodic biofilm of MFCs generating more electricity

**Fig. 4:**
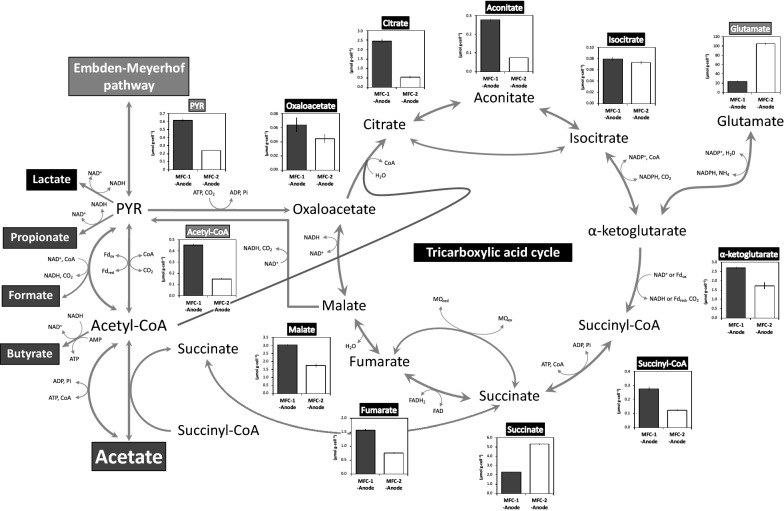
Intracellular concentrations of TCA-related metabolites in microbial cells growing on the MFC anode (MFC-1-anode, black bars; MFC-2-anode, white bars). Metabolite concentrations (µmol g-cell^−1^) were measured after 30 days (MFC-1) and 90 days (MFC-2) of operation. Error bars indicate the standard deviation. Shown are the metabolic flows of organic acids (formate, acetate, propionate, butyrate, and lactate), the flux from acetyl-CoA to pyruvate, as observed in *G. sulfurreducens* [36], and glutamate [51]. CoA, coenzyme A; FAD, flavin adenine dinucleotide; PYR, pyruvate; Fd, ferredoxin; MQ, menaquinone; Pi, phosphoric acid

**Fig. 5:**
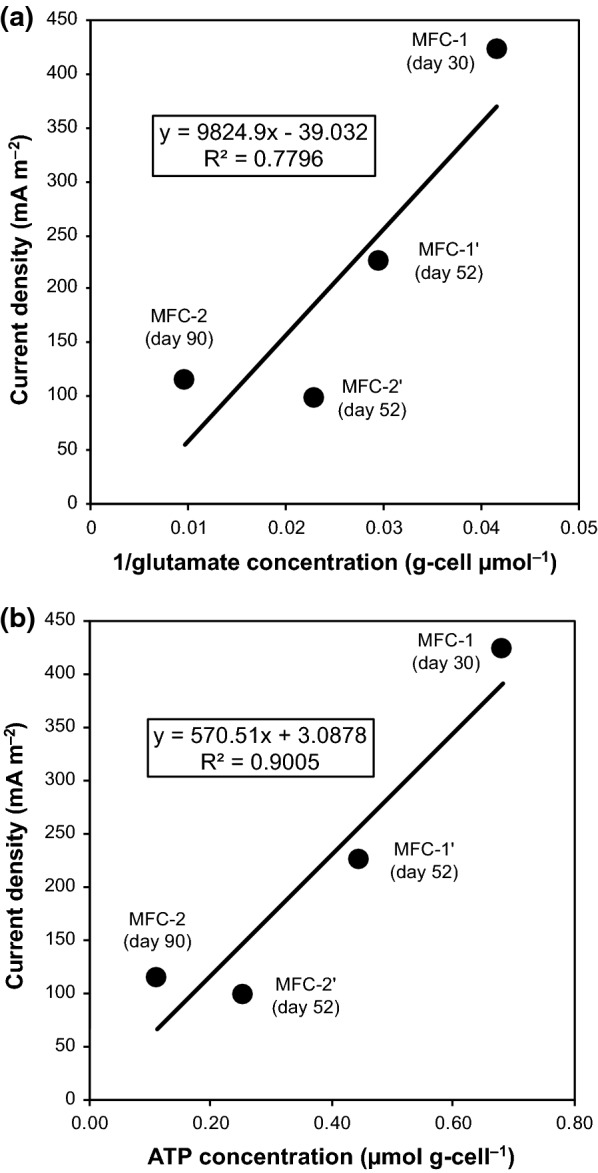
Correlation between current density and **a** inverse intracellular glutamate concentration or **b** intracellular ATP concentration in anodic biofilms of four MFCs. A solid line and its equation indicate the best-fit linear relationship

Metabolomics enables the evaluation of intracellular metabolite levels even in a complex microbial community [30]. Here, the intracellular metabolites in the microbial community on the anodic electrodes were analyzed at the end of MFC operation. The concentrations (µmol g-cell^−1^) of most intracellular metabolites related to the TCA cycle (oxaloacetate, citrate, aconitate, isocitrate, α-ketoglutarate, succinyl-CoA, fumarate, and malate) were higher in the anodic biofilm of MFC-1 than in MFC-2 (Fig. 4), as well as in that of MFC-1′ compared to MFC-2′ (Additional file 3). Interestingly, the intracellular concentration of glutamate correlated negatively with current density in all four MFCs (Figs. 4, 5a).

### Increased ATP/ADP and decreased NADH/NAD^+^ correlate with more electricity generated

**Fig. 6:**
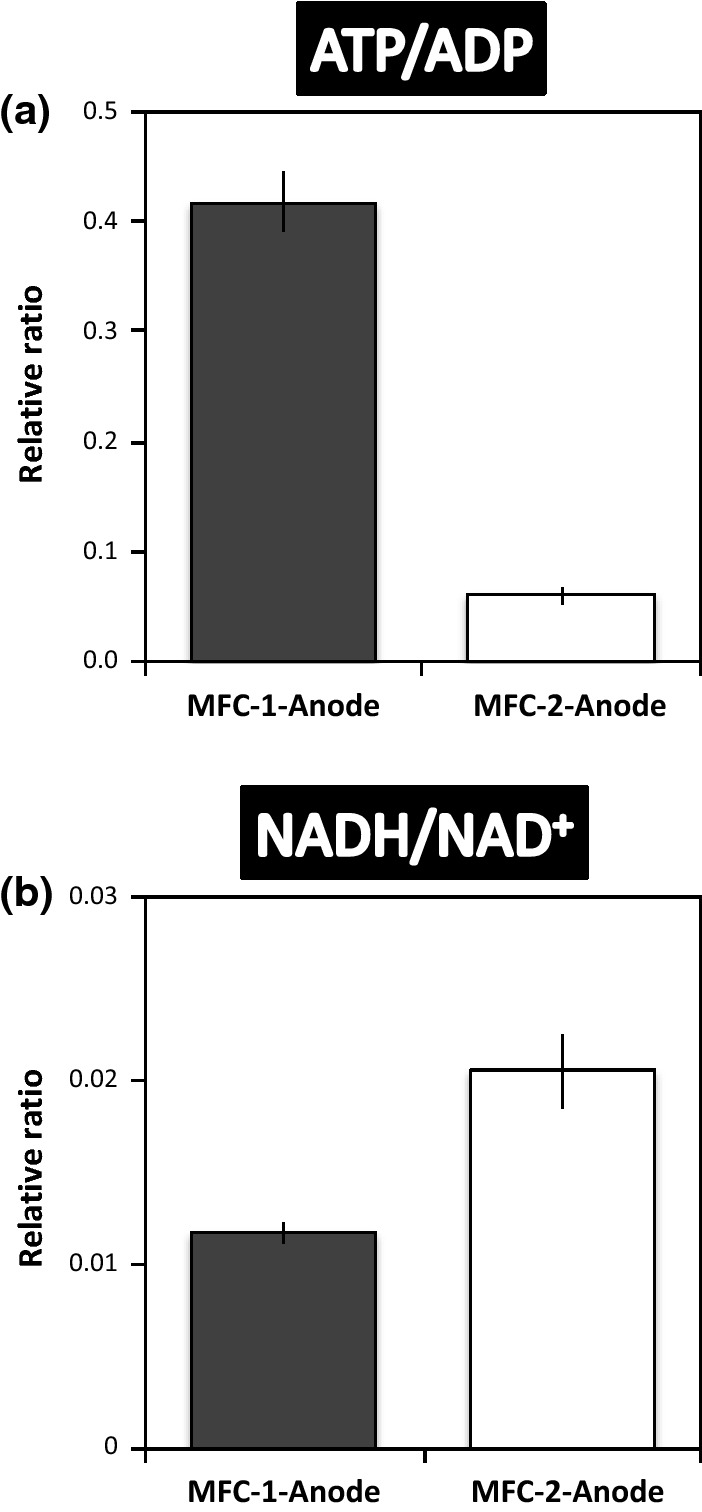
Relative ratios of **a** ATP/ADP and **b** NADH/NAD^+^ in microbial cells growing on MFC anodes (MFC-1-anode, black bars; MFC-2-anode, white bars). Values were calculated based on the amounts of these intracellular metabolites

Our previous metabolomic studies of *G. sulfurreducens* and mixed microbial communities on the anodic biofilm of MFCs operated at different pH conditions showed that the intracellular ATP/ADP ratio increased as electricity generation increased [13, 14]. Here, the intracellular ATP/ADP ratio increased (Student’s *t* test, *P *= 0.001) and NADH/NAD^+^ ratio decreased (Student’s *t* test, *P *= 0.012) in the anodic biofilm of MFC-1, compared to that of MFC-2 (Fig. 6). The same tendency was observed also in the anodic biofilms of MFC-1′ and MFC-2′ (Additional file 4). Intracellular ATP levels correlated well with current density (Fig. 5b). The concentration of intracellular NADPH and NADP^+^ was below the detection limit in this study.

## Discussion

Our results suggest that limited biofilm formation and predominance of *Geobacter* sp. in complex microbial communities on the surface of the anode resulted in relatively more electricity being generated by the MFC. This means that excessive growth of biofilm containing microorganisms and probably extracellular polysaccharides prevented efficient electricity generation. High current generation with a small anodic biofilm of a complex microbial community corresponded with the previous report for single species, *G. sulfurreducens*, that very thick anodic biofilms accumulated inactive cells in the inner layer, resulting in high diffusion resistance and decreased electrochemical activity [31]. Therefore, low biomass on the anode appears to be a key parameter for improving electricity generation. Among the measured metabolites, we found that glutamate was higher in the MFC with lower electricity generation but higher biomass. A positive correlation between intracellular glutamate and biomass should not be surprising, because glutamate is one of the most abundant metabolites in bacterial cells and stands at the intersection between catabolism and anabolism, and between carbon and nitrogen metabolism. Moreover, intracellular glutamate is high when nutrient supply is abundant [32]. Here, we show a negative correlation between intracellular glutamate level and electricity generation, further confirming how the latter benefitted from reduced biomass. Indeed, efficient electricity generation was attained if the intracellular glutamate concentration on the anode was set to 33.9 μmol g-cell^−1^ or less (Additional file 5).

The observed positive correlation between electricity generation and relative abundance of *Geobacter* sp. in the anodic biofilm can be explained by the greater power output of this species compared to a mixed microbial community [14, 33, 34]. *Geobacter* cells oxidize acetate, an electron donor, to carbon dioxide via the TCA cycle to generate NADH or NADPH [35, 36]. NADH or NADPH dehydrogenase transfers electrons from NADH or NADPH to the respiratory chain, allowing *Geobacter* cells to generate ATP via respiration [37, 38]. In addition, several periplasmic and outer-membrane cytochromes are involved in electron transfer out of the cell to the electrode [35]. Here, activation of the TCA cycle, ATP generation, and NADH consumption were all occurring in the anodic biofilm as electricity generation increased, reflecting a progressive rise in *Geobacter* sp. within the mixed microbial community. Fermentative bacteria, *Bacteroides* sp., *Clostridium* sp., and *Lactococcus* sp., found in our anodic biofilm have been often observed in other anodic biofilms of MFCs [39, 40]. *Desulfovibrio* sp. sulfate-reducing bacteria have been found in the anodic biofilms of MFCs treating sulfate-rich water [41] and *Paludibacter* sp. have been found in the anodic biofilms of MFCs fed with sucrose [42]. These bacteria form symbiotic associations in the anodic biofilm that result in the degradation of organic compounds in the substrate to by-products such as acetate [39].

Previously, Meng et al. [36] proposed that, in the case of *G. sulfurreducens*, directing the carbon flux toward extracellular electron transfer rather than biomass synthesis would be beneficial for the conversion of acetate to electricity. In addition, the previous studies reported that the relative abundance of *Geobacter* cells increased in the anodic biofilm as the current density increased in MFCs fed with acetate [8, 43]. However, it should be noted that this is the case for a single strain and an acetate substrate, whereas a complex microbial community fed a complex substrate such as starch which may behave differently. In our study, acetate was supplied to *Geobacter* by other bacterial strains capable of decomposing complex substrates. Reports suggest that there is competition between exoelectrogens, such as *Geobacter*, and acetoclastic methanogens over the use of acetate, even though exoelectrogens have a kinetic advantage when anode availability is not limited [44]. In the MFC-2 anodic biofilm, which generated less electricity, the acetoclastic methanogen *Methanosarcina* sp. was highly abundant, possibly as a result of a higher affinity for acetate than *Geobacter* cells. A relatively high biomass of the MFC-2 anodic biofilm would be suitable for distributing methanogens in the inner position of the biofilm and promoting methanogenic growth, as methanogenic activity predominates in the inner layer, where the oxidation-reduction potential is low [45]. In contrast, *Methanosarcina* sp. was equally scarce in the anodic biofilms of both MFC-2′ and MFC-1′. Reportedly, *Lactococcus lactis* can convert acetate to acetyl-CoA using acetate kinases [46, 47]. The relative abundance of *Lactococcus* sp. increased in the anodic biofilm of MFC-2′ compared to that of MFC-1′, suggesting that they surpassed *Geobacter* sp. in the utilization of acetate. Excessive biofilm formation on the anode likely weakens the competitive advantage of *Geobacter* cells on acetate. Thus, it will be effective to inhibit excessive biomass formation on the anodic electrode forcibly, to achieve higher electricity generation. This might be attained by cleaning anodic biofilm to keep the biofilm thin [48] or introducing oxygen to the anodic biofilm to suppress methanogens [49].

## Conclusions

Electricity generation by MFCs varied in spite of using the same operational conditions and reactor configuration. A detailed 16S rRNA gene sequencing and metabolomic analysis of the complex microbial community in the anodic biofilm revealed that a decrease in intracellular glutamate and microbial biomass correlated with an increase in electricity generation. Therefore, suppressing excessive biofilm formation on the anodic electrode during MFC operation is one of the keys to improving electricity generation. The latter appears to have benefited also from an increase in the relative abundance of *Geobacter* sp., which could use acetate for current production.

## Additional files

**Additional file 1.** Summary of 16S rRNA gene sequencing data and α-diversity values (Faith’s Phylogenetic Diversity and Shannon’s index) in MFCs after 52 days of operation.

**Additional file 2.** Species-level composition of archaea and bacteria in the anodic biofilm (Anode) and fermentation broth (Broth) of MFC-1′ and MFC-2′.

**Additional file 3.** Intracellular concentrations of metabolites related to the TCA cycle in microbial cells growing on the MFC anode (MFC-1′-Anode, black bars; MFC-2′-Anode, white bars).

**Additional file 4.** Relative ratios of (a) ATP/ADP and (b) NADH/NAD+ in microbial cells growing on MFC anodes (MFC-1′-Anode, black bars; MFC-2′-Anode, white bars). Values were calculated based on the amounts of these metabolites.

**Additional file 5.** Relationship between current density and intracellular glutamate concentration in the anodic biofilm of MFCs.

## Abbreviations

C.E.: Coulombic efficiency
COD: chemical oxygen demand
GC-Q-MS: gas chromatography-quadrupole-mass spectrometry
LC-QqQ-MS: liquid chromatography-triple-stage quadrupole-mass spectrometry
MFC: microbial fuel cell
NADH: nicotinamide adenine dinucleotide
NADPH: nicotinamide adenine dinucleotide phosphate
OD_600_: optical density at 600 nm
OTUs: operational taxonomic units
*R*_ext_: external resistance
SS: suspended solids
TCA: tricarboxylic acid
