# Supplementary material for: Less biomass and intracellular glutamate in anodic biofilms lead to efficient electricity generation by microbial fuel cells
Source: Biotechnol Biofuels. 2019 Apr 1;12:72. doi: 10.1186/s13068-019-1414-y (PMC6442422; doi:10.1186/s13068-019-1414-y)
Supplement: Supplementary file 1 — Additional file 1. Summary of 16S rRNA gene sequencing data and α-diversity values (Faith’s Phylogenetic Diversity and Shannon’s index) in MFCs after 52 days of operation. [file 13068_2019_1414_MOESM1_ESM.pdf]

Additional file 1

Summary of 16S rRNA gene sequencing data and α-diversity values (Faith's Phylogenetic Diversity and Shannon's index) in MFCs after 52 days of operation.

	Read counts	Observed OTUs	Faith's Phylogenetic Diversity	Shannon's index
**MFC-1′-Anode**	95,534	305	21.6	5.32
**MFC-2′-Anode**	110,110	381	26.5	5.68
**MFC-1′-Broth**	59,060	203	16.5	4.73
**MFC-2′-Broth**	96,756	257	18.2	4.72

OTUs, operational taxonomic units.
