# Supplementary material for: Less biomass and intracellular glutamate in anodic biofilms lead to efficient electricity generation by microbial fuel cells
Source: Biotechnol Biofuels. 2019 Apr 1;12:72. doi: 10.1186/s13068-019-1414-y (PMC6442422; doi:10.1186/s13068-019-1414-y)
Supplement: Supplementary file 2 — Additional file 2. Species-level composition of archaea and bacteria in the anodic biofilm (Anode) and fermentation broth (Broth) of MFC-1′ and MFC-2′. [file 13068_2019_1414_MOESM2_ESM.pdf]

# Additional file 2

**Species-level composition of archaea and bacteria in the anodic biofilm (Anode) and fermentation broth (Broth) of MFC-1′ and MFC-2′.**

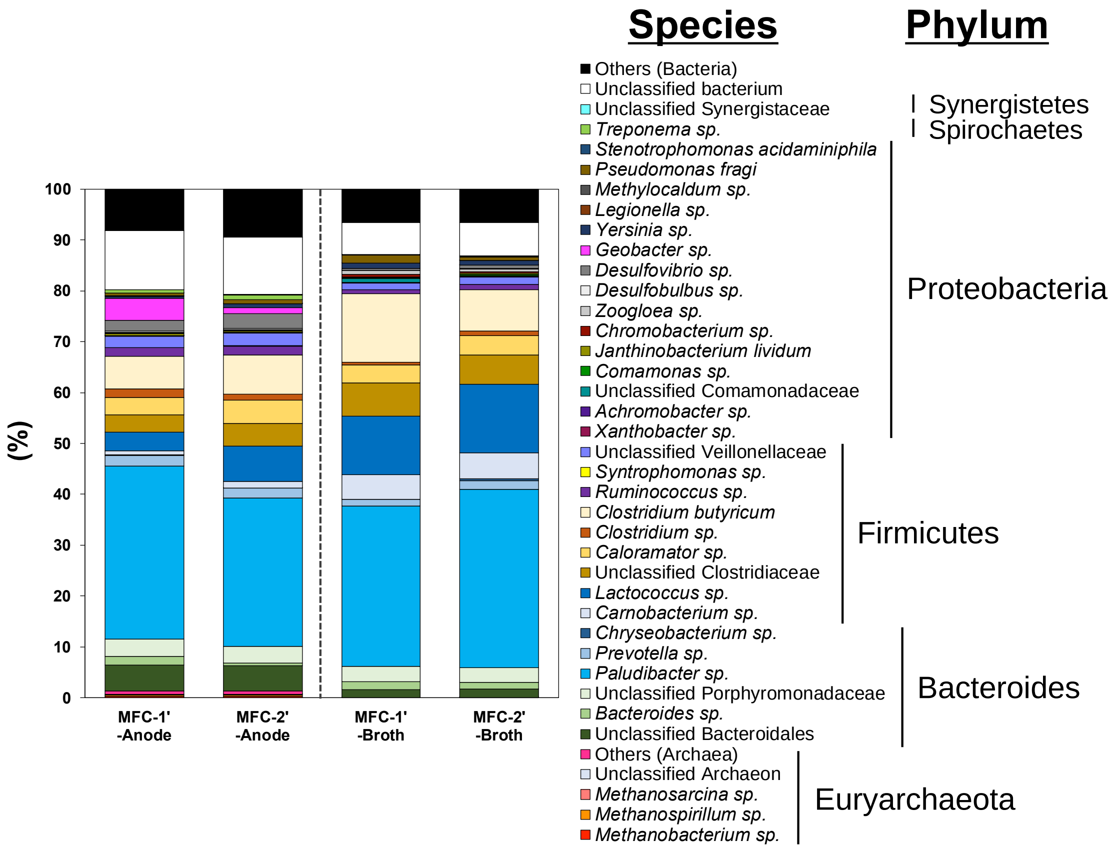


Samples were obtained from anodic biofilms and fermentation broths after 52 days of operation. Not clustered (<99% similarity) and low-abundance (<1.0%) sequences were included in Unclassified and Others, respectively.
