# Supplementary material for: Less biomass and intracellular glutamate in anodic biofilms lead to efficient electricity generation by microbial fuel cells
Source: Biotechnol Biofuels. 2019 Apr 1;12:72. doi: 10.1186/s13068-019-1414-y (PMC6442422; doi:10.1186/s13068-019-1414-y)
Supplement: Supplementary file 3 — Additional file 3. Intracellular concentrations of metabolites related to the TCA cycle in microbial cells growing on the MFC anode (MFC-1′-Anode, black bars; MFC-2′-Anode, white bars). [file 13068_2019_1414_MOESM3_ESM.pdf]

# <u>Additional file 3</u>

**Intracellular concentrations of metabolites related to the TCA cycle in microbial cells growing on the MFC anode (MFC-1′-Anode, black bars; MFC-2′-Anode, white bars).**

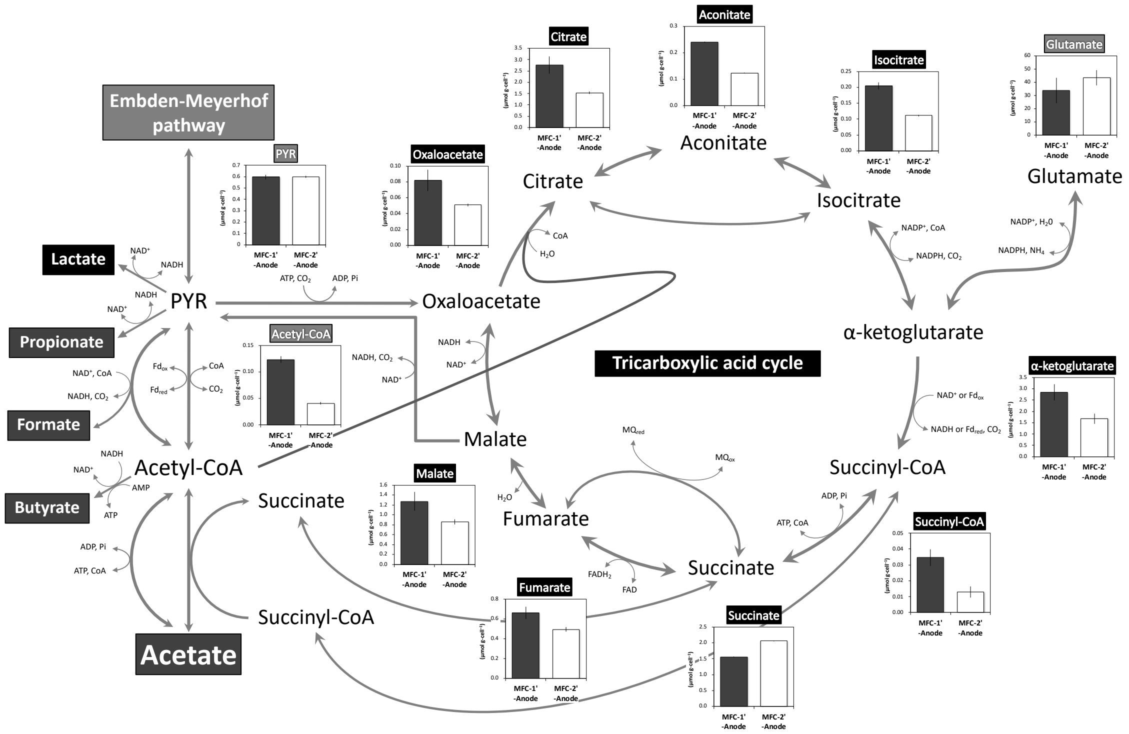


Metabolite concentrations (μmol g-cell$^{-1}$) were measured after 52 days of operation. Error bars indicate the standard deviation. Shown are the metabolic flows of organic acids (formate, acetate, propionate, butyrate and lactate), the flux from acetyl-CoA to pyruvate, as observed in *G. sulfurreducens* (Mahadevan et al. 2006), and glutamate (Reitzer 2003). CoA, coenzyme A; FAD, flavin adenine dinucleotide; PYR, pyruvate; Fd, ferredoxin; MQ, menaquinone; Pi, phosphoric acid.
