# Supplementary material for: Less biomass and intracellular glutamate in anodic biofilms lead to efficient electricity generation by microbial fuel cells
Source: Biotechnol Biofuels. 2019 Apr 1;12:72. doi: 10.1186/s13068-019-1414-y (PMC6442422; doi:10.1186/s13068-019-1414-y)

Additional file 4

Relative ratios of (a) ATP/ADP and (b) NADH/NAD$^+$ in microbial cells growing on MFC anodes (MFC-1′-Anode, black bars; MFC-2′-Anode, white bars). Values were calculated based on the amounts of these metabolites.

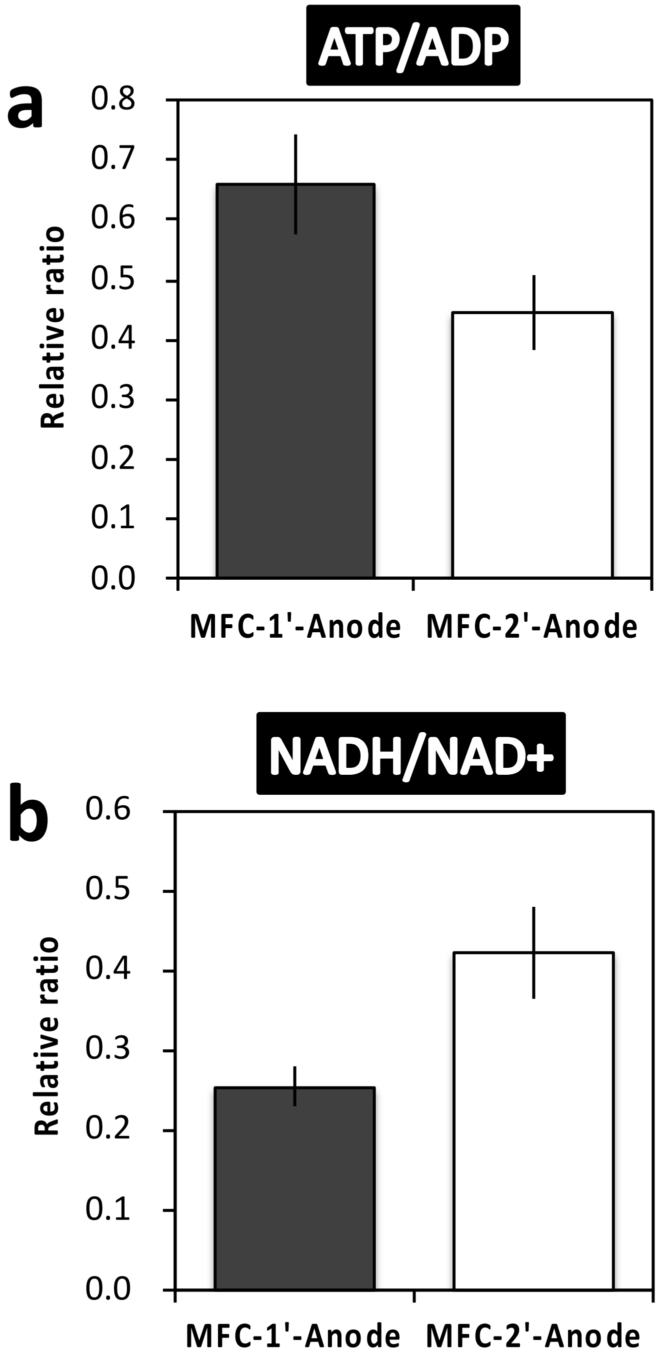

Supplement: Supplementary file 4 — Additional file 4. Relative ratios of (a) ATP/ADP and (b) NADH/NAD+ in microbial cells growing on MFC anodes (MFC-1′-Anode, black bars; MFC-2′-Anode, white bars). Values were calculated based on the amounts of these metabolites. [file 13068_2019_1414_MOESM4_ESM.pdf]
