# Supplementary material for: Less biomass and intracellular glutamate in anodic biofilms lead to efficient electricity generation by microbial fuel cells
Source: Biotechnol Biofuels. 2019 Apr 1;12:72. doi: 10.1186/s13068-019-1414-y (PMC6442422; doi:10.1186/s13068-019-1414-y)

Additional file 5

Relationship between current density and intracellular glutamate concentration in the anodic biofilm of MFCs.

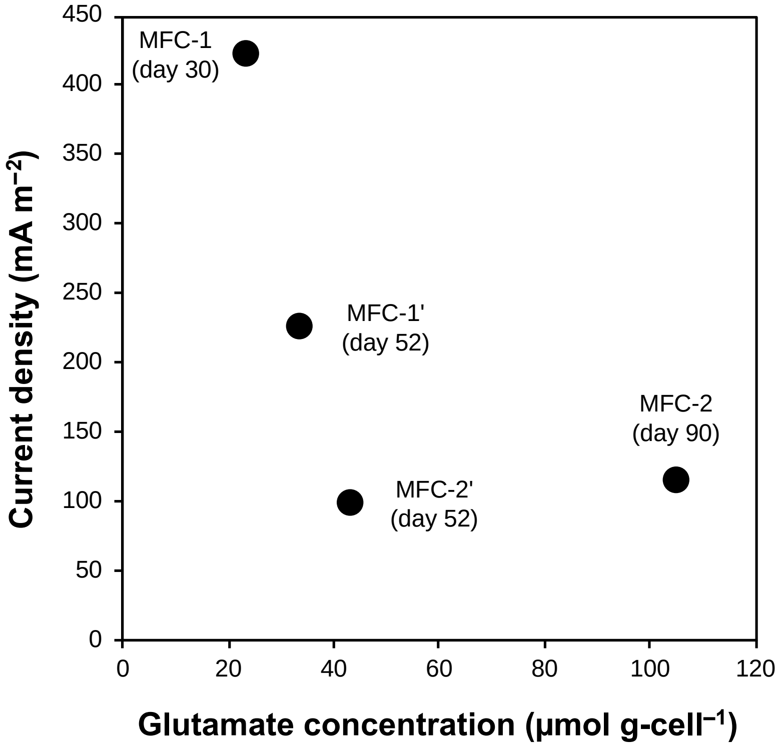

Supplement: Supplementary file 5 — Additional file 5. Relationship between current density and intracellular glutamate concentration in the anodic biofilm of MFCs. [file 13068_2019_1414_MOESM5_ESM.pdf]
